# Male Breast Cancer: An Updated Review of Patient Characteristics, Genetics, and Outcome

**DOI:** 10.1155/2024/9003572

**Published:** 2024-03-23

**Authors:** Vidhu Shekhar Khare, Farhanul Huda, Subhasis Misra, Kanmatha Reddy Amulya, Nirmal Raj, Summi Karn, Somprakas Basu

**Affiliations:** ^1^Department of General Surgery, All India Institute of Medical Sciences, 249203, Rishikesh, India; ^2^Department of Surgical Oncology, BayCare Health System, Department of Medical Education, University of South Florida, Tampa, USA

## Abstract

Male breast cancer (MBC) is a rare entity, underrepresented in population studies and clinical trials, resulting in management of MBC to be informed by current research on female breast cancer (FBC). A literature review was conducted by accessing relevant articles on 2 databases, by searching keywords “male breast cancer”. A total of 29 articles from year 2011 to 2022 were selected for this review. The authors found that male breast cancer generally occurs later in life with higher stage, higher grade, and more estrogen receptor (ER) positive tumours. Most of the studies noted the mean age for MBCs at the time of presentation as >60 years. Risk factors for male breast cancer include family history, obesity, lower physical activity, and syndromes like the Klinefelter syndrome. Positive family history is much higher in MBC compared to FBC (30.9 vs. 18.4%). BRCA 2 cancers constitute a higher proportion compared to FBCs. A lot of genetic mutations have been observed. Some show promise to assess disease-specific survival and proliferative rate like *TWIST1* and *RUNX3*, among others. MBCs usually present with a palpable lump in central region, with a bigger size and chance of nodal involvement and metastasis compared to FBCs. They are mostly infiltrating ductal type and hormone receptor positive, with worse histological grade. Treatment usually follows the same principles as FBCs (systemic therapy, surgical excision, and radiotherapy), with poorer prognosis to same treatment approach, possibly owing to its advanced stage at presentation. This is a rare entity which requires further research to ascertain need for different management approach than FBCs.

## 1. Introduction

Male breast cancer (MBC) is a rare entity, accounting for less than 0.5% of all male cancer diagnoses made annually [1]. Even as its incidence is increasing, it has been relatively underrepresented in population studies and clinical trials. This forces the management of MBC to be informed by current research on female breast cancer (FBC), even though MBCs have been observed to have significantly different tumour biology. More than 90% are ER positive, and they are less likely to express HER2, with higher rates of androgen receptor expression [2]. This review seeks to consolidate recent research work conducted on MBCs to form a current perspective and guide future direction of research.

## 2. Materials and Methods

Literature review was conducted by accessing relevant articles on 2 databases, http://www.pubmed.gov and http://www.doaj.com, by searching keywords “male breast cancer”. All articles with research on MBC, as well as review articles on MBCs, were included. A total of 29 articles from year 2011 to 2022 were selected for this review.

### 2.1. Epidemiology

Male breast cancer generally occurs later in life with higher stage, higher grade, and more estrogen receptor (ER) positive tumours.

Most of the studies have noted that the mean age for male breast cancers (MBCs) at the time of presentation is >60 years, ranging from 62 years [3] to 68 years [4]. A retrospective study of 152 MBC and 304 FBC patients in the period 1990-2014 by Zhao et al. observed that MBC occurred usually in people aged >65 years, as well as 10 years later than FBC patients [5]. Another retrospective study of 39 patients of male breast cancer by Soliman and Hetnał noted the mean age at diagnosis to be 59 years [6].

A national survey conducted in Taiwan of 578 male and 100,915 female breast cancers noted that males tend to be older, have more comorbidities, and receive less chemo- and radiotherapy [7]. An observational retrospective study of 53 MBCs by Herrero et al. observed that 77% of the patients were former smokers and 17% had history of alcoholism [4]. A SEER-based analysis of 1704 MBCs and 225,417 FBCs from 2010 to 2016 by Fang et al. observed that MBCs had older age and a higher rate of Black race [8].

The observations reflect that there should be a higher index of suspicion of MBCs in older age groups, and then, the patients might have more comorbidities and increase risk of mortality owing to advanced age.

### 2.2. Risk Factors

Risk factors generally associated with the development of MBCs include family history, obesity, and lower physical activity, along with syndromes like the Klinefelter syndrome [9].

Soliman and Hetnał noted that 7.7% of the patients had a positive family history [6]. Herrero et al. observed that 23% of the patients had a family history of breast or ovarian cancer. BRCA positive (BRCA 2) mutations were present in 26% of patient overall and 42% in patients with a family history of breast or ovarian cancer [4].

The retrospective study of 152 MBC and 304 FBC patients in the period 1990-2014 observed that positive family history is much higher in MBC compared to FBC (30.9 vs. 18.4%) [5]. HPV infection is not a known association with MBC. A study assessing 27 male breast cancers and 27 gynaecomastia specimens failed to demonstrate any human papillomavirus (HPV) infection in either of them [10].

The association with family history has been cited in these studies, but it is not mentioned which degree of relatives was taken into consideration. Based on the observations, genetic testing should be strongly encouraged in patients with diagnoses of MBC.

### 2.3. Genetics

A study assessing promoter methylation in male breast cancers by Deb et al. observed that *GSTP1* was the most commonly methylated gene, followed by *RASSF1A* out of the 10 gene panels studied. BRCA2 breast cancers had a higher gene methylation rate compared to the other groups [11]. *RARβ* hypermethylated cases had a higher percentage of Paget's disease, as well as larger tumour size. *RUNX3* hypermethylation was associated with IC-NST histological type, and *RASSF1A* hypermethylation was associated with the coexistence of high-grade DCIS. *APC* hypermethylation was associated with older age at presentation, and *MAL* hypermethylation was inversely associated with age. Worse disease-specific survival was observed with high average methylated index and *TWIST1* hypermethylation, and it was also associated with shorter survival in BRCA 2 male breast cancers [11].

Rizzolo et al. in their study assessing 597 male breast cancers with 1022 controls found out that CYP17A1 and CYP1B1 polymorphisms do not contribute significant risk of male breast cancer [12].

A cross-sectional study of 70 MBCs using next-generation sequencing analysis with a panel of 94 cancer predisposition genes by Tedaldi et al. observed c.4964_4982del and c.5266dupC pathological variants in BRCA 1 gene and c.1238delT, c.1813delA, c.3195_3198delTAAT, c.5073dupA, and c.6039delA pathological variants in BRCA 2 gene [13]. Pathogenic variants observed in other genes were c.8319_8323dupTGTCC variant in ATM gene, the c.1100delC variant in CHEK2 gene, the c.3538_3541delGAAG in the EGFR gene, the c.1110_1116delCATGCAG variant in BAP1 gene, the c.73A>T variant in PALB2 gene, and the c.181_182delCT in RAD51C gene. Note that BAP1 gene variant was novel [13].

Zelli et al. did an RNA sequencing analysis of 63 MBCs and found out that DNA damage and repair genes BARD1, BRIP1, and XRCC2 and cell cycle regulation genes FOXM1 and AURKA were significantly upregulated in germline-mutated MBCs compared to nonmutated MBCs. Higher proliferation and HER2 signalling module scores were observed in germline-mutated MBCs [14].

An analysis of 614 MBCs from German Consortium for Hereditary Breast and Ovarian Cancer (GC-HBOC) in period 1964-2018 by Rolfes et al. revealed that 27.7% of patients had a pathological variant in BRCA 1 or 2, with a notably higher proportion of BRCA 2 pathological variants. Most frequent pathological variant in BRCA2 was c.1813dup, whereas c.5266dup was the most common pathological variant in BRCA1 carriers. The patients were predominantly ER+ and PR+ (88.2%). Including HER2 status, most patients were ER+, PR+, and HER2- (77.7%) [15].

Some of these mutations may help us to prognosticate specific subtypes, although further research is necessary to evaluate the clinical significance of these mutations. Ongoing advances in targeted therapy may result in more effective and selective strategies for selective MBCs.

### 2.4. Clinical Presentation

Most common presentation in MBCs is a lump in areolar/nipple region [3, 5, 6, 16, 17]. Only one study found that the tumour was associated with gynaecomastia in 4% of cases [3]. The median tumour size was noted around 42.8 ± 135.7 mm in an analysis of 19,795 MBCs from 2014 to 2017 in National Cancer Database (NCDB) [18].

Soliman and Hetnał noted in his study of 39 MBCs that all the patients presented with breast mass, and around 30% had axillary mass, and two-thirds was T3/T4 tumours [6]. A retrospective study of 22 male breast cancers by Rebaza et al. noted that skin involvement was present in 32.5% of cases [19].

Özkurt et al. noted in their retrospective study of 57 MBCs that 41.5% of the patients had T4 tumours and 65.6% of the patients had N1 disease with <4 lymph node positivity [16].

The SEER data analysis of MBCs from 2010 to 2015 by Xie et al. observed that 7% of cases had distant metastasis at the time of presentation, who had a higher proportion of <60-year-old patients, were grade III-IV, and were more likely to receive chemotherapy. The most common single site of metastasis was the bone (41%), followed by the lung, liver, and brain. Most of the metastatic patients presented with metastasis of single organ (58%) [20]. The study by Herrero et al. of 53 MBCs observed that 41% of patients presented in stage II (most common), followed by stage 0-I (19%) and stage III (19%). 57% had nodal infiltration, out of which 23% of patients 4 or more nodes involved. 14% of the patients presented with distant metastases [4].

While nodal involvement is frequently seen in MBC, there is some discrepancy in studies regarding overall incidence of nodal metastases in MBC versus FBC. A retrospective study comparing 45 male with 75 matched female breast cancers (stages I-III) from 1994 to 2014 by Liu et al. observed that most of the MBC presented at stage II (53.3%, followed by stage I, 26.7%, and stage III, 20%), and fewer MBCs had nodal involvement at the time of diagnosis compared to female breast cancers (FBCs) (46.7% compared to 57.3%) [21]. The retrospective study of 152 MBC and 304 FBC patients in the period 1990-2014 by Zhao et al. observed that lymph nodal invasion was more frequently present in MBCs (44.1% vs. 34.2% in FBCs) [5].

Fang et al. in their SEER-based analysis of 1704 MBCs and 225,417 FBCs from 2010-2016 observed that rate of regional lymph node metastases was much higher in MBCs (40.08% vs. 27.69% in FBCs), and the LN metastatic rate was higher in hormone-/HER2+ subtype. MBCs also had higher incidence of distant metastases at the time of diagnosis (5.93% vs. 4.12% in FBCs), with bone being the most common metastatic site (85.29%). Hormone-/HER2- had the highest distant metastatic rate in MBC (21.26%) [8]. Elimimian et al., in their analysis of MBCs and FBCs in National Cancer Database (NCDB), observed that male patients presented with later clinical staging and exhibit worse disease grade and worse comorbidity scores. MBCs also exhibited more central/nipple disease compared to upper outer quadrant disease in FBCs [1].

Another SEER database analysis of 169,278 breast cancer patients from 2010 to 2014 by Yao et al. observed that MBCs had larger tumour size and higher rate of nodal involvement and organ metastases. 47.91% of MBCs presented with tumours in central portion [17].

Broadly, MBCs present with a palpable lump, usually in central region, and have bigger size, higher chance of nodal involvement, and higher probability of presenting with metastases (usually to bone) compared with FBCs.

### 2.5. Histology

Infiltrating ductal carcinoma is the most common histological type present in MBCs [1, 3, 5, 6, 17].

A study of 127 MBCs in Morocco by Bourhafour et al. observed that 96% of cases were infiltrating ductal carcinoma. 82% of cases were grade II or III (Scarff-Bloom-Richardson). ER and PR hormone receptors were positive in 64% of the patients [3]. Soliman and Hetnał noted that 80% of the patients were hormone receptor positive. Infiltrating ductal carcinoma was the most common type (92.3%), and most of the tumours were grade 2 (76%) [6].

A SEER database study from 1990 to 2010 of 2677 ER+ MBCs by Wei et al. observed that PR-negative patients were younger, more likely to be African American, present with higher grade and larger tumour size and with more lymph nodal involvement. Compared to PR-positive patients, they received more chemotherapy. There was no difference between ER+/PR+ and ER+/PR- MBCs in terms of laterality, surgery, or radiation therapy [22]. Özkurt et al. observed in their retrospective study of 57 MBCs that 92% of the patients had invasive breast carcinoma. Receptor status of 50 patients was achieved, and in those, 92% was ER+, 86% was PR+, and 10% was Her2+. 80% of the tumours were of luminal A subtype, 12% was luminal B, and 6% was basal-like [16].

Herrero et al. observed in their study of 53 MBCs that 89% of cancers were infiltrating, 42% had vascular invasion, and 64% had Ki67 proliferation index >14%. 91% of the MBCs were luminal type, and 6% were HER2 positive [4]. A retrospective analysis of 19,795 male breast cancer patients from 2014 to 2017 by Konduri et al. observed that most of the tumours were differentiated (49.4%), primary lesions (75.1%), and invasive (87.2%). Most of the patients expressed ER (91.7%) and/or PR (82.9%) [18].

The SEER-based analysis by Fang et al. observed that MBCs had higher rate of ductal histology, hormone positive status, poorer tumour differentiation, and later TNM stage [8]. It is rare to have other histological variants such as tubular or mucinous carcinoma which have a better prognosis compared to ductal type [23]. Another SEER database analysis of 169,278 breast cancer patients from 2010 to 2014 by Yao et al. observed that MBCs were more likely to present with advanced grades, and 84.86% of MBCs were invasive ductal carcinoma (compared to 77.81% in FBCs). MBCs were also more likely to express hormone receptor positive (91.63% vs. 72.46% in females) and lower percentage of HER2 negative (11.4% vs. 14.68% in females) [17].

There is correlation between expression of HER2 with high histological grade and tumour size. There is no correlation between expression of EGFR, GLUT1, and CAIX in MBC with any clinicopathological feature, in contrast to female breast cancer. Only expression of EGFR is significantly associated with loss of ER*α* and PR expression [24].

Therefore, molecular imaging in male breast cancer may be best done by a panel of CD44v6, EGFR, HER2, IGF1-R, and GLUT1, supplemented by FGFR2 and CAXII which increases the sensitivity for male breast cancer [24].

A retrospective study of 22 MBCs by Rebaza et al. noted that 45% of male breast cancer specimen expressed androgen receptors (AR) which were also associated with low clinical size, ER-positive status, and PgR-positive status [19].

A retrospective study of 720 MBCs studying hepatocyte growth factor (HGF) and CXCL12 expression in tumour tissue by Qiu et al. observed median CXCR4 expression per tumour as 50% in cytoplasm and 11% in nucleus. The median *H*-score (<20% difference in percentage) for CXCL12 expression was 100 both in cytoplasm and in nucleus. The median *H*-score for HGF expression was 106 [25].

We can summarise our observations as MBCs are mostly infiltrating ductal carcinoma and hormone receptor positive and have worse histological grade compared to FBCs. This highlights the importance of hormonal therapy in MBCs and the need for early diagnosis, as it might be managed more effectively if diagnosed before becoming advanced or metastatic. They have lower rate of expression of Her2 neu as compared to FBCs, with Her2 positive and hormone positive/Her2 negative having worse prognosis. Sensitivity for MBCs in molecular imaging can be increased by adding FGFR2 and CAXII to a panel of CD44v6, EGFR, HER2, IGF1-R, and GLUT1.

### 2.6. Difference from Female Breast Cancer

In a comparative study between male and female breast cancer in Netherlands by Vermeulen et al., it was observed that IGF-R was more frequently expressed in MBC, while MET and FGFR2 were less frequently expressed. Expression of EGF and Her2 was comparable in both MBC and FBC. There was significantly lower expression of CD44v6 and higher expression of CAXII MBC compared to FBC [24].

Hung et al. observed in their national survey conducted in Taiwan of 578 male and 100,915 female breast cancers that the standardised incidence rate (SIR) for a second nonbreast primary cancer was higher compared to female patients (HR 3.01) [7].

The SEER data analysis of MBCs from 2005 to 2010 by Liu et al. observed that male patients were older and more likely to be Black, diagnosed at an advanced stage, LN positive, ER positive, PR positive, and less likely to have surgery [26].

Xie et al. observed in their SEER data analysis of MBCs from 2010 to 2015 that comparing metastasis in male and female breast cancer, incidence of liver metastases was significantly lower in male breast cancer (3% vs. 8%) in terms of single organ metastasis. In terms of multiple organ metastases, bone and liver metastases in male breast cancer were lower, but bone and lung metastases were higher than female breast cancer, as the rate of liver mets is lower in MBC [20].

### 2.7. Treatment

Treatment of localised invasive early MBC usually follows the same principles as in FBCs [9]. But due to presentation of the disease at later stages, along with worse prognostic features, usually the treatment given is more radical than their female counterpart.

Bourhafour et al. reported that 71% of the observed 27 MBCs required radical mastectomy (RM) due to muscular involvement, and 5.5% of the patients were treated by modified radical mastectomy (MRM). 5.5% cases underwent simple mastectomy, and 0.78% cases were treated by lumpectomy. All of them received adjuvant therapy. 66% received radiotherapy, and chemotherapy was given as neoadjuvant therapy to 8% and as adjuvant therapy to 38%. Hormonal therapy was given to 44.8% patients, of which 39.3% patients received tamoxifen alone, 3% received tamoxifen with orchidectomy, and 2.3% underwent orchidectomy alone [3].

The retrospective study of 39 MBCs by Soliman and Hetnał noted that 87.2% of patients underwent MRM. Adjuvant systemic therapy was given to 56.4% of the patients, and radiotherapy was given to 51% of patients. Tamoxifen was given to 31 patients and adjuvant therapy [6]. Özkurt et al., in their retrospective study of 57 MBCs, observed that 77% of patients had to undergo MRM, and 79.2% of the patients underwent axillary lymph node dissection due to clinical node positivity or positive SLNB [16].

Herrero et al. observed that 89% of the patients underwent surgery, 87% of which were mastectomies. 47% of the patients received adjuvant radiotherapy and 85% received endocrine therapy (ET). Of those receiving ET, tamoxifen was used in 89% of cases, the rest receiving aromatase inhibitors. 26% of the patients received chemotherapy [4]. The retrospective analysis of 19,795 MBCs from 2014 to 2017 by Konduri et al. observed that majority of patients underwent surgery (90.6%) but did not receive chemotherapy (63.4%) or radiotherapy (65.9%). 26.7% of the patients received endocrine therapy, and 1.1% of the patients received immunotherapy [18].

The retrospective study by Liu et al. comparing 45 male and 75 matched female breast cancers (stages I-III) from 1994 to 2014 observed that male patients underwent mastectomy more frequently (97.8% of males compared to 6% of females), and of those, fewer received radiotherapy (22.4%) compared to female breast cancers (44.4%). Fewer MBCs received chemotherapy (42.2% compared to 65.3% of females), and there was no significant difference between MBCs and FBCs receiving endocrine therapy. 1 MBC did not undergo mastectomy or lumpectomy (2.2%) [21].

Zhao et al., in their retrospective study of 152 MBC and 304 FBC patients in the period 1990-2014, observed that most of the MBC patients underwent radical mastectomy (84.9%). 73.7% of patients received chemotherapy, and 40.8% of patients received radiation therapy. 38.8% of patients with ER+ tumours received endocrine therapy, which was significantly lower than FBC. None of the MBCs received targeted therapy for HER2 [5].

The SEER-based analysis of 1704 MBCs and 225,417 FBCs from 2010 to 2016 by Fang et al. observed that MBCs had poorer OS than FBCs, making gender an independent prognostic factor for OS [8]. This is likely due to the more advanced stages at diagnosis of MBC.

A retrospective analysis of 71 patients with MBCs treated between 2003 and 2019 by Rolf et al. came to the conclusion that even though postoperative RT is underused in male breast cancers as compared to female, it should be offered according to the guidelines developed for female breast cancers [27].

Another SEER-based analysis of 514 stages I-III MBCs treated between 2010 and 2015 comparing patients who received chemotherapy with patients who did not receive chemotherapy by Li et al. came to the conclusion that adjuvant chemotherapy may be omitted for stage I and IIA MBCs [28].

Zhang et al., in their SEER-based analysis of 6426 MBCs in period 1975-2016, observed that among all the MBCs who died, 42.75% died of breast cancer and 57.3% died of nonbreast cancer causes including cardiovascular disease (CVD) (26.9%), other cancers, and nervous system disease. CVD as the cause of death was more common than breast cancer in age >75 years [29].

These studies demonstrate that MBCs may be managed better if full use of all available therapeutic options is undertaken, including systemic and radiotherapy. The current treatment protocol is not standardised, and despite largely being the same as for FBCs, the excisions are more radical. Discussion of the cases in multidisciplinary treatment boards should be incorporated widely to ensure personalised and effective treatment.

### 2.8. Prognosis

As the disease is rare, there is a significant lacuna of large series studying the significance of prognostic factors, especially molecular subtyping. The study in Morocco observed that local recurrence was seen in 17% of patients, and metastasis was observed in 32% of all cases within the follow-up period of 30 months. Most common site of metastases was the bone (48.7% of all recurrence), followed by the lung (29.3%), liver (17.1%), liver and skin, and pleura and skin (2.4% each) [3].

The retrospective study of 39 MBC patients by Soliman and Hetnał noted that 17.9% of patients had relapse and 5% of the patients had local recurrence. Commonly involved sites were the bone, lung, and liver. Five-year disease-free survival (DFS) was 100% for N0 patients and 44% for N1 patients. Patients with hormone receptor status had significantly higher (88%) DFS as compared to hormone receptor negative patients (25%). Five-year overall survival (OS) of the patients was 84% and was significantly higher in N0 patients than in patients with nodal involvement [6].

Rebaza et al., in their retrospective study of 22 MBCs, noted that cN0 was associated with longer DFS. Younger age and right sided tumours were associated with longer OS. They did not find an association between survival and ER-positive or AR-positive (≥10%) status [19].

The SEER database study of 2677 MBCs by Wei et al. observed that ER+/PR- patients had significantly worse shorter OS and breast cancer-specific survival (BCSS) compared to ER+/PR+ patients [22], corroborated by other studies [26].

Özkurt et al. observed in their retrospective study of 57 MBCs that 1 of the patients had chest wall recurrence in 168^th^ month, 1 had axillary recurrence in 93^rd^ month, and 4 patients had systemic metastasis. Five-year OS, DFS, and DSS rates were 80.75, 965, and 95.6%, whereas 10-year OS, DFS, and DSS rates were 71.6%, 81.9%, and 91.7% [16].

The SEER data analysis of male breast cancer patients from 2010 to 2015 by Xie et al. observed that there was no difference in OS between MBC and FBC patients with distant metastases. Patients ≥ 60 years had a worse prognosis, as well as grades III-IV, triple negative subtype, and those with distant metastases. Patients receiving chemotherapy and surgery had a better prognosis [20].

The observational retrospective study of 53 MBCs by Herrero et al. observed that better OS at 5 years was associated with performance score 0, absence of vascular invasion, Ki67 ≤ 14%, and absence of distant metastases at time of diagnosis [4].

Konduri et al. observed in their retrospective analysis of 19,795 MBCs from 2014 to 2017 that overall survival was lower for MBC. Five-year survival was 75% vs. 85%, and 10-year survival was 56% vs. 71% for male and female breast cancer, respectively. Increasing age and tumour size were independent factors affecting mortality. Tumour stage carried the highest risk of mortality. HER2 expression was also associated with higher mortality [18].

On the contrary, the study by Liu et al. comparing 45 males with 75 matched female breast cancers (stages I-III) from 1994 to 2014 observed that there was no difference in OS and DFS of MBCs compared to FBCs [21]. This may be less representative observation due to smaller sample size of the study.

The study of 720 MBCs studying hepatocyte growth factor (HGF) and CXCL12 expression in tumour tissue by Qiu et al. observed that high HGF expression in tumour cells was an independent predictor for better OS in nonmetastatic setting (13.0 years compared to 7.5 years in HGF low expressing tumours). High expression of CXCL12 in cytoplasm of tumour cells was also associated with better OS [25].

The study by Zhao et al. of 152 MBC and 304 FBC patients in the period 1990-2014 observed that there was no significant difference in local recurrence rates in MBCs and FBCs, but a higher rate of distant metastases was observed in MBCs (47.7 vs. 37.6%). MBCs also had a higher rate of secondary tumour in other organs (16.8% vs. 5.4%). The 5- and 10-year survival of MBCs and FBCs were 74.6% vs. 86.9% and 50.6% vs. 65.7%, respectively. The prognosis was worse for MBCs in luminal subtypes, but similar OS and DFS were present in nonluminal subtypes [5]. The difference in survival could be attributed to the fact that stage for stage overall prognosis is same. However, MBC tends to present at advanced stages and hence a higher likelihood of worse survival.

A SEER database analysis comparing mastectomy with contralateral prophylactic mastectomy (CPM) in MBCs (stages I-III) from 1998 to 2015 by Li et al. made a nomogram to predict 3-year, 5-year, and 8-year breast cancer-specific death (BCSD) and came to the conclusion that CPM was associated with decrease in risk of BCSD in MBCs [30].

The analysis of MBCs and FBCs in National Cancer Database (NCDB) by Elimimian et al. observed that males had significantly worse OS than females. Five-year OS was 72.8% in MBCs compared to 83.4% in FBCs, and 10-year OS was 52.5% vs. 69.1% in FBCs. Unadjusted hazard of early death was 75% higher in males than in females [1].

Another SEER database analysis of 169,278 breast cancer patients from 2010 to 2014 by Yao et al. observed that MBCs had a worse overall OS than FBCs. Tumour location was an independent prognostic marker for both MBCs and FBCs, and medial tumours had a poorer prognosis than both central and lateral location [17].

## 3. Conclusion

The number of studies done on MBCs is meagre compared to FBCs owing to the rare nature of disease. MBCs usually present at a later age, with a central palpable mass, more often in later stages of disease as well as metastases. They are frequently hormone positive, with many new mutations which need further enquiry to determine their role in prognosis and management. Further research into the significance of these mutations and possible targeted therapies is the need of the hour. A panel of CD44v6, EGFR, HER2, IGF1-R, and GLUT1, supplemented by FGFR2 and CAXII can be used to increase sensitivity for MBCs in molecular imaging. They are usually treated with a radical approach, partially owing to their advanced stage at presentation. The rates of adjuvant chemo- and radiotherapy given to MBCs have been lower retrospectively, which should be further enquired into and rectified. Proper deployment of all available treatment strategies, i.e., excision, radiotherapy, systemic therapy, and targeted therapy, should be employed using a multidisciplinary approach. The overall prognosis of MBCs is worse than their female counterparts.

As clear from the current literature review, male breast cancer is an important, albeit rare entity with a significant dearth of multicentric, large studies. Most of the data is retrieved during studies of female breast cancer, and only recently, there have been efforts to better understand this entity. Further research is necessary to explore if there is a need for different management approaches.
